# Free Radical Production Induced by Nitroimidazole Compounds Lead to Cell Death in *Leishmania infantum* Amastigotes

**DOI:** 10.3390/molecules29174041

**Published:** 2024-08-26

**Authors:** Julia Andrés-Rodríguez, María-Cristina González-Montero, Nerea García-Fernández, Estefanía Calvo-Álvarez, María-Yolanda Pérez-Pertejo, Rosa-María Reguera-Torres, Rafael Balaña-Fouce, Carlos García-Estrada

**Affiliations:** 1 Departamento de Ciencias Biomédicas, Facultad de Veterinaria, Universidad de León, Campus de Vegazana s/n, 24071 León, Spain; jandrr01@estudiantes.unileon.es (J.A.-R.); magom@unileon.es (M.-C.G.-M.); ngarf@unileon.es (N.G.-F.); myperp@unileon.es (M.-Y.P.-P.); rmregt@unileon.es (R.-M.R.-T.); 2 Department of Pharmacological and Biomolecular Sciences, University of Milan, 20133 Milan, Italy; estefania.calvo@unimi.it; 3 Instituto de Biomedicina (IBIOMED), Universidad de León, Campus de Vegazana s/n, 24071 León, Spain

**Keywords:** *Leishmania infantum*, visceral leishmaniais, neglected tropical diseases, nitroaromatic compounds, drug repurposing, pretomanid, fexinidazole, trypanothione reductase

## Abstract

*Leishmania infantum* is the vector-borne trypanosomatid parasite causing visceral leishmaniasis in the Mediterranean basin. This neglected tropical disease is treated with a limited number of obsolete drugs that are not exempt from adverse effects and whose overuse has promoted the emergence of resistant pathogens. In the search for novel antitrypanosomatid molecules that help overcome these drawbacks, drug repurposing has emerged as a good strategy. Nitroaromatic compounds have been found in drug discovery campaigns as promising antileishmanial molecules. Fexinidazole (recently introduced for the treatment of stages 1 and 2 of African trypanosomiasis), and pretomanid, which share the nitroimidazole nitroaromatic structure, have provided antileishmanial activity in different studies. In this work, we have tested the in vitro efficacy of these two nitroimidazoles to validate our 384-well high-throughput screening (HTS) platform consisting of *L. infantum* parasites emitting the near-infrared fluorescent protein (iRFP) as a biomarker of cell viability. These molecules showed good efficacy in both axenic and intramacrophage amastigotes and were poorly cytotoxic in RAW 264.7 and HepG2 cultures. Fexinidazole and pretomanid induced the production of ROS in axenic amastigotes but were not able to inhibit trypanothione reductase (TryR), thus suggesting that these compounds may target thiol metabolism through a different mechanism of action.

## 1. Introduction

The trypanosomatid protozoan parasite *Leishmania infantum* is the etiological agent of visceral leishmaniasis in the European Mediterranean basin and the Americas (syn = *Leishmania chagasi*), whereas in Africa and Asia *Leishmania donovani* is the species responsible for this disease. Leishmaniasis has been included by WHO, along with African (sleeping sickness) and American trypanosomiasis (Chagas disease), within the group of neglected tropical diseases (NTDs) caused by trypanosomatids that are widely spread in the tropical and subtropical areas of the planet [1,2,3]. *Leishmania* is transmitted by the bite of sandflies of the genera *Phebotomus* and *Lutztomya* and presents two main morphological forms: promastigote (motile flagellated form) in the sandfly vector, and amastigote (intramacrophage non-motile form) in the mammalian host [4]. Clinical manifestations depend on the *Leishmania* species infecting the mammalian host, thereby giving rise to three main types of leishmaniasis: cutaneous, mucocutaneous, and visceral [5], the latter being the most severe form affecting the poorest populations in developing countries [6], with an estimated incidence of 50,000 to 90,000 new cases (only 25–45% cases reported) and at least 5710 deaths in 2019 [7].

Due to the lack of a specific vaccine for the human disease, chemotherapy is the only treatment available for leishmaniasis and is based on scarce and obsolete drugs that have plenty of undesired side effects, and whose overuse has promoted the appearance of resistance [8,9]. Pentavalent antimonials (Sb^V^; Glucantime and Pentostam), although they still represent the first-line treatment for different types of leishmaniasis in many endemic countries, exhibit serious side effects, such as cardiotoxicity and pancreatitis, require long-term intramuscular injections, and have given rise to the emergence of resistant strains [9,10,11]. Amphotericin B (Amp B), formulated either as deoxycholate or liposomal (AmBisome) is very effective, but it requires slow-infusion intravenous administration in hospitals due to its poor oral bioavailability, is unstable at the high temperatures of endemic countries, and shows adverse effects, such as nephrotoxicity, hypokalemia, and myocarditis [12,13,14]. The only oral drug approved for the treatment of different forms of leishmaniasis is miltefosine [14,15]. However, despite its high efficacy, this drug is embryotoxic, thus preventing its use in pregnant women, and after several years of use, a loss of efficacy has been reported [16,17,18,19]. Finally, paromomycin (an aminoglycoside antibiotic), due to its limited efficacy as monotherapy, is used in parenteral formulations in combination with Sb^V^, miltefosine, and AmBisome [9,20,21].

In this scenario, there is an urgent need to develop new natural or synthetic drugs against *Leishmania* [22,23]. One of the strategies followed to reach this goal is drug repurposing (repositioning), i.e., the identification of new antileishmanial compounds from already known drugs [24]. Nitroaromatic compounds have emerged as interesting molecules in drug discovery campaigns for repositioning drugs against trypanosomatids [25,26]. Nitroaromatic rings (nitro functional group attached to benzene, a heterocycle such as furan or imidazole, or a bicycle), are privileged pharmacophores of many antimicrobials in clinical use, including antifungal and antiparasitic drugs [27]. These compounds act as prodrugs and require nitroreductase (NTR)-mediated activation to exert cytotoxic effects [26,27,28,29]. Although the exact mechanism of action has not been fully characterized for nitroaromatic compounds, it is assumed that reductive bioactivation and generation of reactive intermediates are responsible for the overall effect [30]. These intermediates are a source of free radicals, which are likely to interact with various biochemical pathways of both the host and parasite, inducing cell death in the absence of suitable scavengers. In this scenario, different redox-active antiparasitic drugs have been described and enzymes catalyzing vital redox reactions represent potential targets for drug development [31]. For example, it is well-known that some nitrofurans target the unique thiol metabolism of trypanosomatids behaving as inhibitors of trypanothione reductase (TryR), a key enzyme in the maintenance of redox balance in these parasites [26,32,33,34].

**Figure 1:**
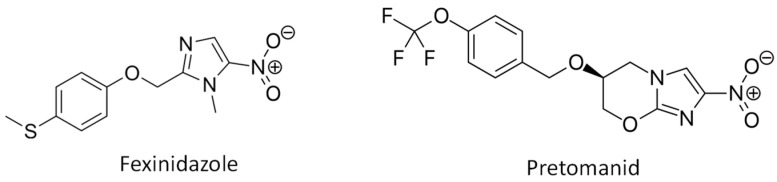
Chemical structure of the nitroimidazoles fexinidazole and pretomanid.

Nitroaromatic compounds are currently used in the clinical practice for the treatment of trypanosomatid-borne diseases, including benznidazole (nitroimidazole), nifurtimox (nitrofuran), both used for the treatment of Chagas disease for more than five decades [35,36,37], and fexinidazole (nitroimidazole), the latter being approved more recently for the oral treatment of the stages 1 and 2 of African trypanosomiasis [26,38]. In addition, different nitro derivative molecules are currently being tested against different trypanosomatids, and nitroimidazoles, which are broad-spectrum antimicrobial drugs with antibacterial activity, represent a promising source of antitrypanosomal and antileishmanial agents [26,39]. As mentioned above, fexinidazole (Figure 1) is currently used for the treatment of African trypanosomiasis and has been also tested against *Leishmania* both in vitro and in vivo with good results [40,41]. Recently, oral self-emulsifying systems containing fexinidazole have provided successful results in vivo, thus representing promising oral alternatives for the treatment of visceral and cutaneous leishmaniasis [42,43]. On the other hand, the R enantiomers of the antitubercular 4-nitroimidazo-oxazine pretomanid (PA-824) (Figure 1) have provided good antileishmanial effects in vitro and in vivo [44].

In this article, the in vitro efficacy of fexinidazole and pretomanid has been assessed in a biotechnological platform consisting of *L. infantum*-engineered amastigotes that express the gene encoding the near-infrared fluoresce protein (iRFP) as a biomarker of cell viability, thereby validating a high-throughput screening (HTS) for drug discovery in a 384-well format. In addition, reactive oxygen species (ROS) production and TryR activity have been determined in order to provide some insights into the putative mechanism of action of these two nitroimidazole compounds.

## 2. Results

### 2.1. In Vitro Efficacy of Nitroimidazole Compounds on L. infantum-iRFP Amastigotes

The antileishmanial effect of fexinidazole and pretomanid was tested against *L. infantum*-iRFP, an engineered strain constitutively expressing the iRFP [45] (see Section 4). Both axenic and intramacrophage amastigotes were obtained from mice infected with *L. infantum*-iRFP promastigotes, as described in Section 4, and the infrared fluorescence emitted at 700 nm by the iRFP produced by living amastigotes was used as a direct measurement of the parasite viability, as previously characterized [45].

**Figure 2:**
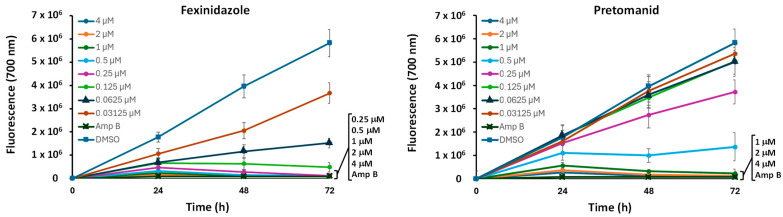
Evolution of fluorescence emission by axenic amastigotes of *L. infantum*-iRFP throughout the culture time from 0 h to 72 h in the presence of different concentrations (from 4 μM to 0.0312 μM) of either fexinidazole or pretomanid, 0.1% (*v*/*v*) DMSO (negative control), and 18 µM Amp B (positive control). The fluorescence emitted at 0 h was subtracted from the rest of the values. Results show the mean values ± SD of three independent experiments with at least three technical replicates each.

In an initial experiment, we analyzed the evolution of cell viability based on fluoresce emission by *L. infantum*-iRFP axenic amastigotes at 0 h, 24 h, 48 h, and 72 h after the addition of different concentrations of either fexinidazole or pretomanid. DMSO (solvent of nitroimidazole compounds) at a concentration of 0.1% (*v*/*v*) was used as a negative control, whereas amphotericin B (18 µM) was added as a positive control. Graphics in Figure 2 show that concentrations above 0.125 μM fexinidazole and 0.5 μM pretomanid produced a decrease in fluorescence emission from 24 h until 72 h, thus confirming the antileishmanial effect of these molecules in the engineered strain.

**Figure 3:**
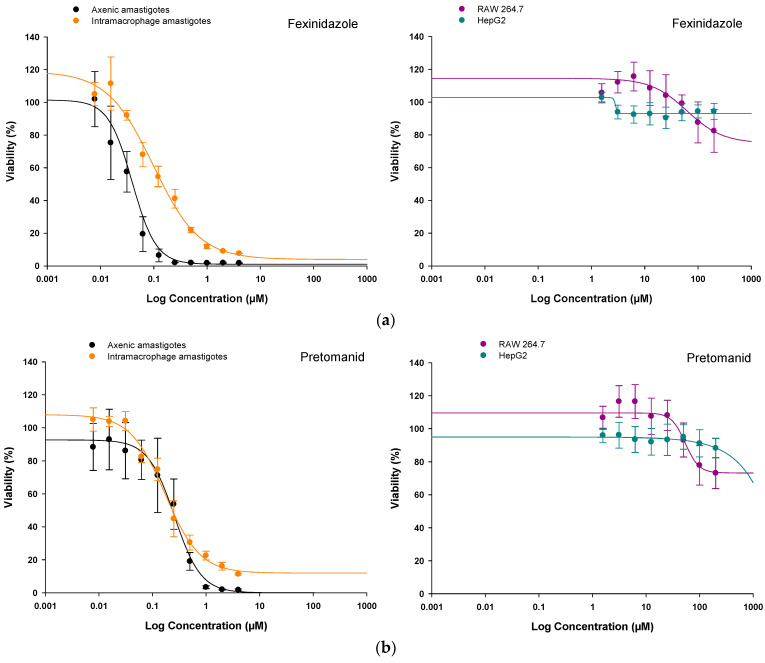
Dose-response curves adjusted with the Sigma Plot 10.1 statistical package showing the effect of (**a**) fexinidazole and (**b**) pretomanid on *L. infantum*-iRFP axenic and intramacrophage amastigotes (**left** panels), and HepG2 and RAW 264.7 cells (**right** panels). Graphs were prepared with viability data obtained from cells after 72 h of incubation in the presence of different concentrations of these compounds: from 4 μM to 0.0078 μM with one-half serial dilutions for parasites, or from 200 μM to 1.56 μM with one-half serial dilutions for mammalian cells. The *y*-axis represents the percentage of cell viability relative to the negative control, while the *x*-axis, in logarithmic scale, represents the concentration (μM) of the different nitroimidazole molecules and amphotericin B. Results show the mean values ± SD of three independent experiments with at least three technical replicates each.

Once the antileishmanial effect of fexinidazole and pretomanid on axenic amastigotes was confirmed, we also tested these two compounds on intramacrophage amastigotes. Since the antileishmanial effect is clearly observed after 72 h in the presence of the nitroimidazoles, we selected this time point to represent the percentage of viability versus several concentrations of each nitroimidazole in dose-response curves, considering that 100% viability is represented by the fluorescence emitted by the amastigotes treated with DMSO (0.1% *v*/*v*) and 0% viability is represented by amastigotes treated with Amp B (18 µM). Curves (Figure 3) were non-linearly adjusted with the Sigma Plot 10.1 statistical package, thus providing EC_50_ values in the low μM or nM range that were higher for intramacrophage amastigotes than for axenic amastigotes (Table 1 and Table 2).

As observed in Table 1 and Table 2, fexinidazole showed the highest potency in axenic amastigotes, whereas pretomanid was the best antileishmanial compound against intramacrophage amastigotes.

### 2.2. In Vitro Safety of Nitroimidazole Compounds on HepG2 and RAW 264.7 Mammalian Cells

The cytotoxicity of fexinidazole and pretomanid was tested in the mammalian cell lines HepG2 (a human cell line used to test the systemic toxicity of the compounds) and RAW 264.7 (a mouse cell line used to test the toxicity in macrophages), which were incubated in the presence of different concentrations of these compounds (from 200 μM to 1.56 μM with one-half serial dilutions). Cell viability was assessed using the alamarBlue^TM^ staining method (Invitrogen, Waltham, MA, USA), taking as 100% viability the fluorescence emitted by the cells treated with 0.1% (*v*/*v*) DMSO (negative control) and as 0% viability the signal provided by those cells treated with 0.1% (*v*/*v*) H_2_O_2_ (positive control). The percentage of viability was plotted versus several concentrations of each nitroimidazole in comparative dose-response curves, which were non-linearly adjusted with the Sigma Plot 10.1 statistical package. These curves (Figure 3) showed the low cytotoxicity of these molecules for HepG2 and RAW 264.7 mammalian cells. Consequently, the CC_50_ could not be calculated for the range of the concentrations tested (1.56 μM to 200 μM), thus providing a CC_50_ value of >200 μM (Table 1 and Table 2). Using the EC_50_ and CC_50_ values, the Selectivity Index (SI) for each nitroimidazole compound against axenic and intramacrophage amastigotes was calculated. As shown in Table 1, SI values of more than three orders of magnitude were obtained regarding axenic amastigotes, and fexinidazole showed the highest SI. Regarding intramacrophage amastigotes, pretomanid provided the best score. However, SI values were lower than those provided with axenic amastigotes, although still high, showing numbers of more than two orders of magnitude (Table 2).

### 2.3. Induction of ROS Production in L. infantum by Fexinidazole and Pretomanid

**Figure 4:**
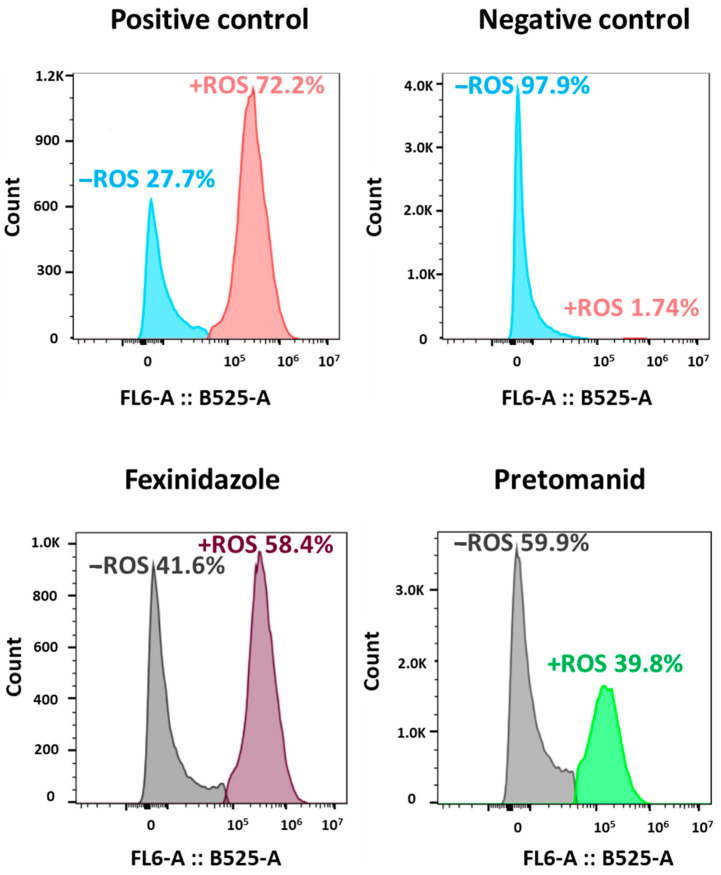
Representative flow cytometry graphs showing ROS production by the axenic amastigote cultures of *L. infantum*-iRFP stained with DCFH-DA after the addition of 0.01% (*v*/*v*) H_2_O_2_ (positive control), 0.03% (*v*/*v*) DMSO, 0.04 μM fexinidazole, and 0.28 μM pretomanid. Histograms represent the distribution of fluorescence intensity for FL6-A: B525-A. Results show the mean values of two independent experiments with three technical replicates each.

Once the in vitro efficacy and safety of the three nitroimidazole compounds were tested, we decided to analyze the ability of these molecules to induce ROS production in an attempt to provide some hints into the mechanism of action responsible for the loss of parasite viability. For this purpose, cultures of *L. infantum*-iRFP axenic amastigotes were treated with 0.04 μM fexinidazole and 0.28 μM pretomanid (the EC_50_ obtained in previous experiments for each nitroimidazole compound) for 3 h and labeled with 2′,7′-dichlorofluorescein diacetate (DCFH-DA) for ROS production assessment. Amastigote cultures were also treated with H_2_O_2_ (0.01% *v*/*v*) as positive control or with 0.03% (*v*/*v*) DMSO as negative control. Flow cytometry graphs showed distinct peaks corresponding to the stressed and unstressed populations, and an induction of ROS production by the nitroimidazole compounds compared to the negative control. Fexinidazole was the compound inducing more ROS production, giving rise to a percentage of the stressed population of 58.4 ± 1.5%, whereas pretomanid provided values of 39.8 ± 1.2% (Figure 4).

### 2.4. In Vitro Inhibitory Effect of Fexinidazole and Pretomanid on L. infantum TryR

**Figure 5:**
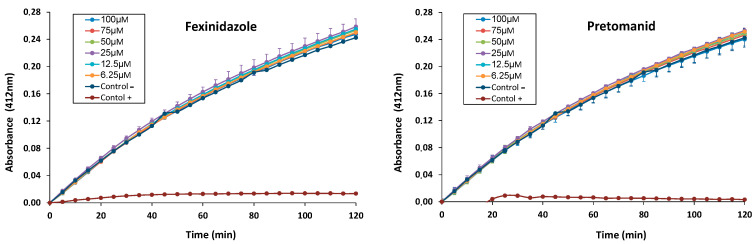
Time-dependent inhibition of TryR activity with a fixed concentration of T[S]_2_ (0.075 mM) and NADPH (0.20 mM). Thioridazine (0.1 mM) was used as a positive control. Fexinidazole and pretomanid were tested at concentrations ranging from 6.25 μM to 100 μM. The calculated specific enzymatic activity for the negative control (without inhibitor) was 4.83 × 10^−3^ μmol/mg · min. (ΔA/t = ɛ · d · c; ɛ = 14,150 M^−1^ cm^−1^; d = 0.34 cm). Results show the mean values ± SD of two independent experiments with three technical replicates each.

In order to correlate the oxidative stress induced by fexinidazole and pretomanid with the mechanism of action, the potential inhibitory role of these molecules on TryR was assessed. Therefore, different concentrations of these nitroimidazoles were added to *L. infantum*-iRFP protein extracts, and the time-dependent inhibitory effect at saturating concentrations of oxidized trypanothione (T[S]_2_) and NADPH (0.075 mM and 0.20 mM, respectively). As shown in Figure 5, neither fexinidazole nor pretomanid had any inhibitory effect on *L. infantum* TryR at concentrations of up to 100 μM, unlike thioridazine, a well-known inhibitor of TryR, which completely inhibited the enzyme at 100 μM. These results indicate that these two nitroimidazoles induce ROS production by targeting metabolic pathways different from thiol metabolism.

## 3. Discussion

Nitroheterocycle-based molecules have provided potent and effective compounds against trypanosomatids-borne diseases, such as Chagas disease and, more recently, sleeping sickness. Benznidazole (a nitroimidazole) and nifurtimox (a nitrofuran) are two oral antichagasic drugs in medical practice, and the nitroimidazole fexinidazole was recently incorporated in the treatment of sleeping sickness in Africa. In addition, despite the potential mutagenic issues of these compounds, a novel nitro derivative DNDi-0690 has been recently introduced in early clinical phases against visceral and cutaneous leishmaniasis, opening a new window of opportunity for these compounds [24,26].

In the present work, we have validated our in-house biotechnological 384-well HTS platform, consisting of an engineered iRFP-emitting *L. infantum* [25], by testing the antileishmanial activity of fexinidazole and pretomanid, two nitroheterocyclic compounds of the nitroimidazole family. Fexinidazole (1-methyl-2-((p-(methylthio)-phenoxy)methyl)-5-nitroimidazole) is an orally administered nitroheterocyclic derivative that has recently been introduced in some African countries, after FDA approval, as the first all-oral therapy for the haemolymphatic and meningoencephalic forms of sleeping sickness [38]. Fexinidazole produced parasite remission in mouse models of visceral leishmaniasis [40], but its therapeutic effect was inconclusive in a Phase II clinical trial [46]. Pretomanid (PA-824) is an orally administered nitroimidazooxazine antimycobacterial agent, which was approved in 2019 by the FDA to treat highly challenging cases of tuberculosis [47], but it also has a strong killing effect against trypanosomas and Leishmania species and has served as a chemical scaffold of many other compounds with antitrypanosomal activity. Pretomanid exists as a mix of stereoisomers R and S, the R enantiomer being more active than the S form against *L. donovani* both in vitro and in vivo [44].

In our HTS platform, both pretomanid and fexinidazole showed a strong antileishmanial effect on both *L. infantum* iRFP axenic and intramacrophage forms with EC_50_ values in low or below μM range, in the same order as those described for a series of 7-substituted 2-nitroimidazooxazine [48], and good selectivity indexes (higher than the antileishmanial drug Amp B [22]) in mouse macrophages and human hepatic cell lines. Unlike pretomanid, fexinidazole antileishmanial potency was 33-fold lower in intramacrophage amastigotes than in axenic parasites, which is in concordance with previous studies [40,49]. It has been described that fexinidazole undergoes rapid oxidation in vivo to fexinidazole sulfoxide and sulfone [50]. Although fexinidazole and its metabolites showed similar potencies against axenic amastigotes of *L donovani*, when tested against intracellular amastigotes, only the sulfoxide and sulfone forms yielded a potency similar to miltefosine, the parent drug providing little activity, thus pointing to the sulfoxide and sulfone metabolites as the therapeutically relevant species. Based on these observations, the discrepancy between the potency of fexinidazole, and that of the sulfone and sulfoxide metabolites against intracellular amastigotes, has been attributed to the inability of the parent drug fexinidazole to enter or accumulate at therapeutic concentrations within the host macrophage, rather than by a differential activity of fexinidazole depending on whether the target parasite is growing as a free axenic form or within the host cell. This is in line with the physicochemical properties of fexinidazole, which has a higher cLogP than its metabolites and, therefore, binds more readily to plasma proteins [40].

The mechanism of action of nitroheterocycle compounds is probably related to the activation of the nitro group by NTR enzymes, thus conforming to the prodrug nature of these compounds. It has been proposed that the antichagasic drug benznidazole shows antiprotozoal activity because of the formation of electrophilic metabolites resulting from the reduction of the nitro group, whose metabolites covalently bind to macromolecules of the parasite and cause cell damage [26,30]. The existence of two classes of NTRs involved in the enzymatic catalysis of nitro groups is well-stated: the oxygen-insensitive NTR-I, which utilizes NAD(P)H and catalyzes two reduction reactions of the nitro group and does not generate ROS, and the oxygen-sensitive NTR-II, which contains FAD or FMN as cofactor and catalyzes just one reduction reaction generating a nitro radical that reacts with oxygen to produce superoxide anions [51]. Although NTR-II activation was initially proposed as the main mechanism of action of nitroheterocyclic compounds, the fact that overexpression of leishmanial and trypanosomal NTR-I increased the sensitivity of these parasites to fexinidazole—and its metabolites—as well as to benznidazole and nifurtimox, indicated that these molecules are essentially activated by type NTR-I [51,52,53]. In addition, an uncommon NAD(P)H-dependent flavoprotein called NTR2 was reported to be involved in the activation of pretomanid and delamanid in *Leishmania* spp. [29,44]. More studies are required to characterize whether one or several NTR enzymes are involved in the activation of fexinidazole and pretomanid in *L. infantum*, since according to our results, fexinidazole and pretomanid at a concentration corresponding to their EC_50_ value gave rise to a large percentage of *L. infantum* amastigotes accumulating free radicals (the effect being stronger for fexinidazole than for pretomanid), which is consistent with an activation mediated by NTR-II. 

These results made us hypothesize whether the increase in ROS production observed in *L. infantum* could be a consequence of the modification in the unique thiol metabolism of this trypanosomatid parasite. Sensitivity to ROS-inducing drugs of trypanosomatids was early proposed by Docampo [54] in 1990, who speculated about the key role played by trypanothione as a free-radicals scavenger in this family of parasites. Since then, a plethora of compounds (reviewed by [31]) have been identified as free-radical-generating drugs in these pathogens. In the absence of catalase, Se-dependent glutathione peroxidase, glutathione reductase, and thioredoxin reductase, trypanosomatids rely on the low-molecular-weight dithiol peptide trypanothione (N1, N8-bis(glutathionyl)spermidine) as a unique antioxidant defense mechanism [34,55,56]. The trypanothione system represents a sophisticated defense mechanism against reactive species, thereby acting in these parasites as the key reductant in a reaction, where the oxidized disulphide trypanothione (TS_2_) is transformed back into the reduced form T(SH)_2_ by TryR in the presence of NADPH as an electron donor [34,57]. TryR has been reported to be the target of some nitrofuran compounds, which cause irreversible inactivation of the enzyme under anaerobiosis, whereas in the presence of O_2_ behave as subversive substrates that do not inactivate the enzyme, but instead, effectively inhibit the enzymatic reduction of trypanothione, causing the production of free radicals and leading to futile consumption of NADPH [34,58]. For example, uncompetitive inhibition patterns have been reported with 5-nitro-2-furoic acid derivatives [32] and with nifuratel [33]. Regarding fexinidazole and pretomanid, they were not able to inhibit TryR in vitro, which suggests that the increased ROS production in the parasites treated with these compounds is mediated by a mechanism different from TryR enzyme inhibition. The other key enzyme in the trypanothione system is trypanothione synthetase, which catalyzes the biosynthesis of trypanothione. An inhibitory role of fexinidazole and pretomanid on this enzyme cannot be ruled out, since molecules of different nature have been reported to target trypanothione synthetase [34]. Alternatively, due to the relevance of trypanothione as an antioxidant molecule, fexinidazole and pretomanid may behave as thiol scavengers, thus leading to an increase in ROS content because of the decrease in trypanothione, as it has previously been reported for the 5-nitroimidazole megazol in *T. cruzi* [59].

In conclusion, our 364-well HTS fluorescent biotechnological platform has been validated, determining the efficacy of fexinidazole and pretomanid against *L. infantum*-iRFP amastigotes. The cytotoxicity effect of these two nitroimidazoles on *L. infantum* parasites is related to the increase in the intracellular ROS levels, although more studies are needed to elucidate the intimate mechanism of action responsible for cell death, which can provide valuable information for the development of new antitrypanosomatid drugs.

## 4. Materials and Methods

### 4.1. Experimental Animals and Ethical Statement

In this work, Balb/c mice infected with iRFP-*L. infantum* were used to obtain axenic amastigotes from bone marrow and intramacrophagic splenic amastigotes. The animals were acquired by Janvier Laboratories (St Berthevin Cedex, France) and maintained in the University of León animal house, under standard housing conditions with free access to feed and water. The animal handling protocols used in this study comply with the Spanish Act (RD 53/2013) inspired by European Union Legislation (2010/63/UE) and were approved by the Junta de Castilla y León under the authorization number OEBA 007-2019.

### 4.2. Nitroimidazole Compounds

Two nitro derivative compounds from the nitroimidazoles family (fexinidazole and pretomanid) were selected to be tested against *L. infantum*-iRFP parasites (see below). They were purchased from MedChemExpress and dissolved in dimethyl sulfoxide (DMSO). Due to the toxicity of DMSO, serial dilutions of each compound were performed so that DMSO concentration never exceeded 0.1% (*v*/*v*) in the culture medium.

### 4.3. Parasites and Mammalian Cell Lines

*L. infantum*-iRFP, a genetically modified strain of *L. infantum* BCN150 (MCAN/ES/96/BCN 150) constitutively expressing the gene encoding the bacteriophytochrome-based infrared fluorescent protein (iRFP) from *Rhodopseudomonas palustris* [60], was previously created in our laboratory [45], and was used in the in vitro efficacy assays. This strain was cultured as free-living promastigotes in Schneider’s insect medium (Sigma-Aldrich, Merck, Darmstadt, Germany) supplemented with 20% (*v*/*v*) fetal bovine serum (FBS) (Gibco, Thermo Scientific, Waltham, MA, USA) and antibiotic cocktail (100 U/mL penicillin and 100 µg/mL streptomycin) (Hyclone, Thermo Scientific, Waltham, MA USA) at 26 °C until mice were infected.

*L. infantum*-iRFP axenic and intramacrophage amastigotes were obtained from six- to eight-week-old female Balb/c mice after 8 to 10 weeks post-inoculation via intraperitoneal with 1.5 × 10^9^ *L. infantum*-iRFP metacyclic promastigotes [33]. Briefly, to obtain axenic amastigotes, the bone marrow cell suspension was extracted from the femur and tibia of infected mice and passed through a 100-µm cell strainer. After centrifuging at 3500 rcf for 10 min at room temperature, cell suspensions were resuspended in medium containing 15 mM KCl; 136 mM KH_2_PO_4_; 10 mM K_2_HPO_4_ ·3H_2_O; 0.5 mM MgSO_4_ ·7H_2_O; 24 mM NaHCO_3_; 22 mM glucose; 1 mM glutamine; 1× RPMI 1640 vitamin mix (Sigma-Aldrich, Merck, Darmstadt, Germany); 10 mM folic acid; 100 mM adenosin; 1× RPMI amino acid mix (Sigma-Aldrich, Merck, Darmstadt, Germany); 5 mg/mL hemin; 25 mM 2-Morpholinoethanesulphonic acid (MES) pH 5.6, supplemented with 10% FBS (Gibco, Thermo Scientific, Waltham, MA, USA); and antibiotic cocktail, and were incubated at 37 °C and with 5% CO_2_. On the other hand, intramacrophage amastigotes were obtained from the spleen of infected mice, which was cut into pieces, treated with 2 mg/mL collagenase D (Merck, Darmstadt, Germany) and passed through a 100-µm cell strainer. After erythrocyte lysis, the splenocyte suspension containing intramacrophage amastigotes was resuspended in RPMI medium (Gibco, Fisher Scientific, Madrid, Spain) supplemented with 20% FBS, 1 mM sodium pyruvate, 24 mM NaHCO_3_, 2 mM L-glutamine, 1× RPMI vitamins, 25 mM HEPES pH 7.2, and antibiotic cocktail.

For cytotoxicity tests on mammalian cells, HepG2 (human hepatocellular carcinome cell line) and RAW 264.7 (murine macrophage cell line) adherent cells were cultured in Dulbecco’s Modified Eagle Medium (DMEM) and RPMI, respectively, both supplemented with 10% FBS and antibiotic cocktail and incubated at 37 °C and 5% CO_2_.

### 4.4. Assessment of In Vitro Cytotoxicity

To test the in vitro antileishmanial effect of selected nitroimidazoles, 40 μL including either 20,000 axenic amastigotes or a number of murine splenocytes naturally infected with *L. infantum*-iRFP representing 150,000 Relative Fluorescence Units (RFU), were added to each well of 384-well black microtiter plates with optical bottom. Next, 40 μL of one-half serial dilutions of each compound, either in the amastigote culture medium (for axenic amastigotes) or in the supplemented RPMI medium (for intramacrophage amastigotes), were added to each well. For both types of experiments, positive controls (Amp B to a final concentration of 18 µM) and negative controls (0.1% (*v*/*v*) DMSO) were included in every plate. Plates were incubated at 37 °C and 5% CO_2_ for up to 72 h. Fluorescence emitted by living cells of *L. infantum*-iRFP was measured in an Odyssey infrared imaging system (Li-Cor, NE, USA). Readings were taken at 0 h (to ensure correct plate loading), 24 h, 48 h, and 72 h to monitor the evolution of cell viability.

In vitro, cytotoxicity of nitroimidazole compounds was assessed in the mammalian cell lines HepG2 and RAW 264.7. To each well of a 96-well microtiter plate, 100 μL including 10,000 HepG2 or RAW 264.7 cells were seeded and incubated for 24 h at 37 °C and 5% CO_2,_ to allow cells to settle down. Subsequently, 100 μL of one-half serial dilutions of each compound diluted either in DMEM (for HepG2 cells) or RPMI medium (for RAW cells) were added to each well. Cellular viability was measured after 72 h of incubation at 37 °C and 5% CO_2_ using alamarBlue^TM^ Cell Viability Reagent (Invitrogen, Fisher Scientific, Inc.), according to the manufacturer’s recommendations. Positive controls, consisting of 0.1% (*v*/*v*) H_2_O_2_, and negative controls, consisting of 0.1% (*v*/*v*) DMSO, were also included in each plate.

All experiments were carried out in triplicate and included at least three technical replicates. The fluorescence emitted by the negative control wells (containing 0.1% (*v*/*v*) DMSO) was adjusted to 100% viability, whereas the fluorescence emitted by the positive control wells, was adjusted to 0% viability. The percentage of viability for each cell line was plotted against each drug concentration, using the nonlinear fit analysis provided by the Sigma Plot 10.1 statistical package, which provided the EC_50_ and CC_50_ values for each compound. The Selectivity Index (SI) was calculated from the CC_50_/EC_50_ ratio.

### 4.5. Analysis of ROS Production by Axenic Amastigotes

Cultures of *L. infantum*-iRFP axenic amastigotes, which were obtained and maintained as described above, were treated with fexinidazole and pretomanid at the EC_50_ calculated in previous experiments. As a positive control of oxidative stress induction, cultures were treated with 0.01% H_2_O_2_ (*v*/*v*), whereas 0.03% (*v*/*v*) DMSO was added to the axenic amastigotes as a negative control. After incubation for 3 h at 37 °C and 5% CO_2_, cells were harvested by centrifugation at 3500 rcf for 10 min, washed in PBS, centrifuged again, and resuspended in PBS. Cells were labeled by incubation with 5 μM 2′,7′-dichlorofluorescein diacetate (DCFH-DA, MedChemExpress, Monmouth Junction, NJ, USA) at 37 °C for 30 min [61]. Following incubation, cells were centrifuged at 3500 rcf for 5 min, washed, and resuspended in PBS. The cell suspension was stored at 4 °C until flow cytometry analysis.

Flow cytometry was performed to measure intracellular ROS levels in a CytoFLEX SRT (Beckman Coulter, Brea, CA, USA). The experiment was carried out in duplicate and included three technical replicates.

### 4.6. Trypanothione Reductase Enzymatic Assay

The *L. infantum* TryR activity was measured in the presence of different concentrations of nitroimidazole compounds according to [33]. Briefly, 1 × 10^10^ *L. infantum*-iRFP axenic amastigotes were washed twice in PBS and lysed with a solution containing 1 mM EDTA, 40 mM HEPES, 50 mM Tris HCl pH 7.5, 2% (*v*/*v*) Triton X-100, and Pierce™ Protease Inhibitors Mini Tablets (ThermoFisher Scientific Inc., Waltham, MA, USA). After incubation on ice for 15 min, cells were vortexed three times for 15 s in the presence of 0.5-mm diameter glass beads (Merck, Darmstadt, Germany). Cell extracts were obtained by centrifugation at 10,000 rcf for 5 min at 4 °C. The enzymatic assay was carried out in 96-well plates, which included 2 μL of different concentrations of the nitroimidazole compounds diluted in DMSO, 28 μL of TryR assay solution containing 0.2 mM NADPH (Alfa Aesar, Fisher Scientific, Inc., Ward Hill, MA, USA), variable concentrations of T[S]_2_ (Bachem, Fisher Scientific, Inc., Bubendorf, Switzerland), 0.075 mM 5,5’-dithiobis(2-nitrobenzoic acid) (DTNB) (Alfa Aesar, Fisher Scientific, Inc.), and 50 mM Tris HCl pH 7.5. To this solution, 50 μL of cell extracts diluted in Tris HCl pH 7.5 (0.43 μg of total protein) were added to initiate the reaction. As controls in the assay, 2.5% DMSO (negative control), the TryR assay solution without T[S]_2_ (blank reaction), and 0.1 mM thioridazine [62] (Medchem Express, Princeton, NJ, USA) in DMSO (positive inhibition control), were used. Enzymatic activity was measured at 412 nm for a period of up to 120 min (with 5 min intervals) at 26 °C in a Varioskan Lux spectrophotometer (Thermo Scientific, Fisher Scientific, Inc.). The experiment was carried out in duplicate and included three technical replicates.

## Figures and Tables

**Table 1:** Results of EC_50_, CC_50,_ and Selectivity Index (SI) for fexinidazole and pretomanid in axenic *L. infantum*-iRFP axenic amastigotes, HepG2, and RAW 264.7 cells. Amp B data is shown as reference antileishmanial compound.

Tested Compound	Axenic Amastigotes		HepG2 Cells		RAW 264.7 Cells		SI^1^	SI^2^
	EC_50_ Values (μM)	*p*	CC_50_ Values (μM)	*p*	CC_50_ Values (μM)	*p*		
Fexinidazole	0.04 ± 0.00	***	>200.00	N/A	>200.00	N/A	>5000.00	>5000.00
Pretomanid	0.28 ± 0.02	***	>200.00	N/A	>200.00	N/A	>714.29	>714.29
Amp B	0.27 ± 0.02	***	69.75 ± 13.69	***	6.70 ± 0.67	***	258.33	24.82

Note: *** *p* ≤ 0.001. N/A: Not applicable. SI^1^ calculated between CC_50_ HepG2 cells and EC_50_ axenic amastigotes. SI^2^ calculated between CC_50_ RAW 264.7 cells and EC_50_ axenic amastigotes.

**Table 2:** Results of EC_50_, CC_50,_ and Selectivity Index (SI) for fexinidazole and pretomanid in intramacrophage *L. infantum*-iRFP amastigotes, HepG2, and RAW 264.7 cells. Amp B data is shown as reference antileishmanial compound.

Tested Compound	Intramacrophage Amastigotes		HepG2 Cells		RAW 264.7 Cells		SI^1^	SI^2^
	EC_50_ Values (μM)	*p*	CC_50_ Values (μM)	*p*	CC_50_ Values (μM)	*p*		
**Fexinidazole**	1.32 ± 0.06	***	>200.00	N/A	>200.00	N/A	>151.52	>151.52
**Pretomanid**	0.66 ± 0.08	***	>200.00	N/A	>200.00	N/A	>303.03	>303.03
**Amp B**	0.32 ± 0.02	***	69.75 ± 13.69	***	6.70 ± 0.67	***	217.97	20.94

Note: *** *p* ≤ 0.001. N/A: Not applicable. SI^1^ calculated between CC_50_ HepG2 cells and EC_50_ intramacrophage amastigotes. SI^2^ calculated between CC_50_ RAW 264.7 cells and EC_50_ intramacrophage amastigotes.

## Data Availability

Data supporting the results presented in this article are included in the manuscript. Further inquiries can be directed to the corresponding authors.
